# Establishment of a neuroendocrine prostate cancer model driven by the RNA splicing factor SRRM4

**DOI:** 10.18632/oncotarget.19916

**Published:** 2017-08-03

**Authors:** Yinan Li, Ruiqi Chen, Mary Bowden, Fan Mo, Yen-Yi Lin, Martin Gleave, Colin Collins, Xuesen Dong

**Affiliations:** ^1^ Department of Urologic Sciences, Vancouver Prostate Centre, The University of British Columbia, Vancouver, Canada

**Keywords:** neuroendocrine prostate cancer, cell/tumor models, SRRM4, RNA splicing

## Abstract

Neuroendocrine prostate cancer (NEPC) is becoming more prevalent as more potent androgen receptor (AR) pathway inhibitors are applied to patients with metastatic tumors. However, there are limited cell and xenograft models currently available, hindering the investigation of signal pathways involved in regulating NEPC progression and the design of high throughput screening assays for inhibitors to treat NEPC patients. Here, we report an NEPC model, LnNE, that is derived from prostate adenocarcinoma cells and has global similarity in transcription and RNA splicing to tumors from NEPC patients. LnNE xenografts are castrate-resistant and highly aggressive. Its tumor take is ∼3-5 weeks and tumor doubling time is ∼2-3 weeks. LnNE expresses multiple neuroendocrine markers, preserves AR expression, but is PSA negative. Its neuroendocrine phenotype cannot be reversed by androgen treatment. LnNE cells grow as multi-cellular spheroids under 2-dimensional culture conditions similar to the NEPC cell line NCI-H660, but have higher proliferation rate and are easier to be transfected. LnNE cells can also adapt to 3-dimensional culture conditions in a 96-plate format, allowing high throughput screening assays. In summary, the LnNE model is useful to study the mechanisms of NEPC progression and to discover potential therapies for NEPC.

## INTRODUCTION

Although new generation androgen receptor (AR) pathway inhibitors (ARPIs) are effective in prolonging the survival of patients with metastatic prostate cancer [1, 2], emerging evidence indicates that a subtype of castrate-resistant prostate cancer, called treatment-induced neuroendocrine prostate cancer (t-NEPC), is becoming more prevalent due to the selection pressure of ARPIs [3–6]. T-NEPC has been reported in up to ∼25% of patients who had received either first- or second-line anti-AR therapies [7] and this rate of occurrence is predicted to rise with the widespread use of ARPIs. Once diagnosed, the average survival of t-NEPC patients with small cell carcinoma is only ∼7 months [8]. Besides systemic chemotherapy regimens, no targeted therapy has yet been approved for t-NEPC patients. This is in part due to the limited NEPC cell and tumor models available for studying molecular underpinnings of t-NEPC progression.

Whole transcriptome sequencing technology applied on prostate tumor samples had identified many genes whose expression were highly correlated with t-NEPC progression [3, 9, 10]. These findings subsequently require experimental validations on whether these genes can drive NEPC progression; and furthermore, whether a driver gene could be used as potential therapeutic target for the disease. These molecular mechanistic studies require cell or xenograft models that have transcriptome, morphology and cellular physiology similar to those of NEPC tumors in patients. Such NEPC models should also be easily manipulated so that gain- and loss-of-function approaches can be applied to dissect signal cascades regulated by the genes of interest. The later requirement is challenging to patient derived xenografts, patient organoids and genetically engineered mouse models. Among in vitro NEPC cell models, the NCI-H660 line is relatively well characterized [11–13]. It is AR negative, expresses high levels of neuroendocrine markers, and grows as suspending cell clusters, a morphology distinct from adherent luminal epithelial cells such as LNCaP or PC3 cells. When grafted in immune comprised mice, the NCI-H660 line can form NEPC xenografts that progress rapidly [9]. However, its slow growth rate in vitro and low efficacy to be transfected by plasmids or siRNA limit its applications. Several LNCaP-derived cell models were reported to acquire neuroendocrine phenotypes when treated with androgen depletion, IL-6, cAMP, hypoxia and radiation [14–18]. However, it has not been established whether these phenotypical alterations are sustainable during prolonged treatments and whether xenografts derived from these cells can be established and show similar gene signature to NEPC globally.

Our previous studies using whole transcriptome analyses of RNA-seq data from two independent patient cohorts [9, 10] have identified an NEPC specific splice signature [4] that is predominantly regulated by the RNA splicing factor, SRRM4. We report that through generating neuronal-specific splice variants of target genes, SRRM4 can induce neuroendocrine phenotypes and neuronal-like cellular morphology in adenocarcinoma (AdPC) cells and transform AdPC cells into NEPC xenografts. These findings suggest that exogenous expression of SRRM4 in adenocarcinoma cells such as LNCaP cells may enable the establishment of t-NEPC cell and xenograft models.

## RESULTS

### RNA splicing and transcription profiles of LnNE P0 cells

Although exogenous SRRM4 can transform LNCaP cells into NEPC xenografts under androgen depletion conditions [4], several questions remain to be answered: 1) whether LNCaP cells overexpressing SRRM4 have global RNA splicing features and transcriptomes similar to those of NEPC in patients; 2) whether the neuroendocrine phenotype is sustainable during prolonged castration treatment, and the transformed LNCaP cells can be developed into an NEPC cell and xenograft model with stabilized NEPC molecular profile and morphology; and 3) whether the AR expression and or AR signaling will be silenced, mimicking NEPC. To address these questions, we applied RNA sequencing to profile the whole genomic transcription in LNCaP cells overexpressing SRRM4 or SRRM4 plus TP53 knockdown [4]. These two cell lines showed very similar phenotypes to each other with 15.2% (908/5925) - 18.1% (1110/6127) genes differentially expressed at the mRNA level (BenjaminiHochberg corrected FDR<0.01) (Supplementary Figure 1A). The differentially expressed genes were not relevant to NEPC as demonstrated by Gene Set Enrichment Analysis (GSEA) using the Beltran 2016 data set (NES=1.10, FDR=0.39) [3] (Supplementary Figure 1B). Since cells with TP53 depletion showed higher proliferation rate and faster tumor establishment rate [4], we therefore named the LNCaP cells with SRRM4 overexpression plus TP53 knockdown as LnNE P0 in the following experiments.

**Figure 1:**
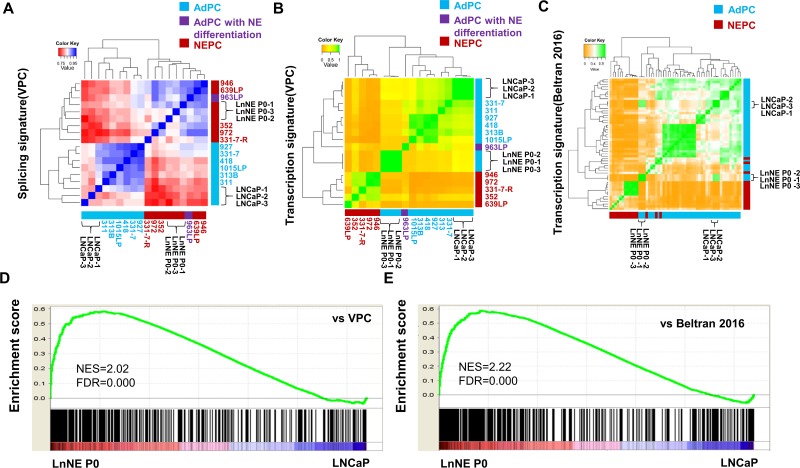
Comparison of RNA splicing and transcriptome of LnNE P0 cells with NEPC **A.** RNA-seq data from LNCaP and LnNE P0 cells were compared with NEPC-specific RNA splicing events from the VPC cohort. Spearman correlation and hierarchical clustering using the *pheatmap* package in R showed the correlations of LnNE P0 splicing signature with that of NEPC patient samples splicing signature of LnNE P0 cells correlated with NEPC. **B.**-**C.** RNA-seq data from LNCaP and LnNE P0 cells were compared with NEPC specific transcripts from the VPC cohort (C) and Beltran 2016 (D). Spearman correlation and hierarchical clustering showed that the LnNE P0 transcriptome was closer to the NEPC transcriptomes compared to those of parental LNCaP cells. **D.**-**E.** GSEA results showed the correlation of the LnNE P0 expression profiles with the top up regulated genes (p-value<0.01) from (D) VPC and (E) Beltran 2016 cohort.

The LnNE P0 cells exhibited splicing profiles distinct from LNCaP cells, but similar to NEPC tumor samples from the VPC cohort containing 6 AdPC, 5 NEPC and 1 AdPC with neuroendocrine differentiation samples [4]. There were 882 different splicing events from 606 genes between NEPC and AdPC samples (BenjaminiHochberg corrected, FDR<0.01) within the VPC cohort. Based on these differentially expressed splicing isoforms, Spearman correlation and hierarchical clustering showed that LnNE P0 cells (n=3 repeats) were similar to NEPC tumors, while LNCaP cells (n=3 repeats) were clustered into the AdPC tumor group (Figure 1A). These results support our previous findings that the NEPC-specific RNA splicing signature is predominantly controlled by SRRM4.

There were 905 genes differentially expressed at the mRNA level (Benjamini-Hochberg corrected, FDR<0.01) between NEPC and AdPC samples from the VPC cohort [4] and 364 genes differentially expressed (Benjamini-Hochberg corrected, FDR<0.01) in the Beltran cohort containing 34 AdPC and 15 NEPC samples [3]. Based on these gene expressions, Spearman correlation and hierarchical clustering showed that both LnNE P0 and LnCaP cells were classified in the AdPC group. However, the transcriptome of LnNE P0 was more similar to the NEPC profile than those of other AdPC samples including the AdPC with neuroendocrine differentiation samples in both cohorts (Figure 1B-1C). In contrast, the transcriptome of LNCaP cells was more distal to the NEPC profile. Since SRRM4 is an RNA splicing factor and the LnNE P0 splicing features are NEPC-like, these results together indicate that SRRM4 mediates RNA splicing programs that drive the transformation of LNCaP transcriptome towards the NEPC transcriptome. Further GSEA assays showed that the expression profiles of the LnNE P0 cells were also significantly correlated with the top ranked genes upregulated in NEPC tumors from both VPC (NES=2.02, FDR<0.0001) and Beltran 2016 (NES=2.22, FDR<0.0001) cohorts (Figure 1D-1E). Together these findings indicate that LnNE P0 cells possess an NEPC specific RNA splicing signature and confirms that SRRM4 drives LnNE transformation to NEPC.

### NEPC progression of LnNE xenografts

**Figure 2:**
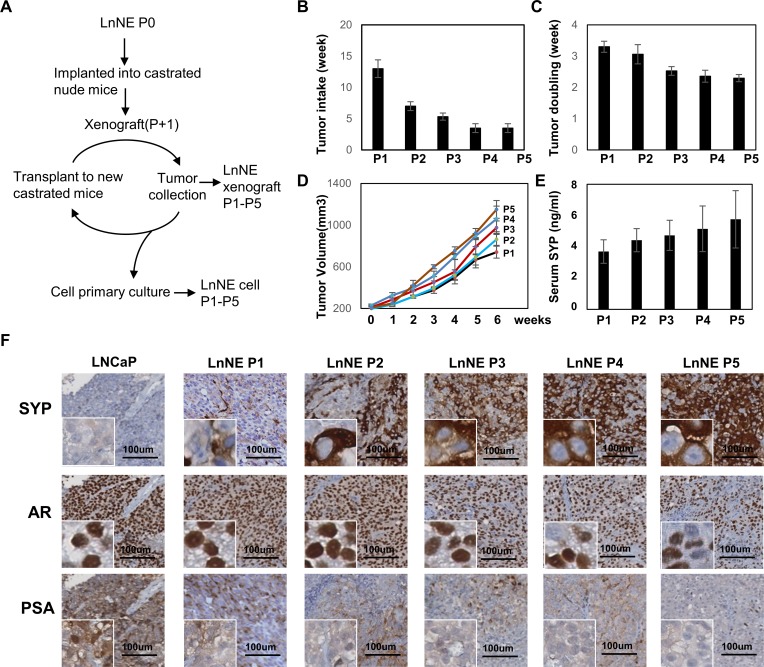
Establishment of LnNE neuroendocrine prostate cancer xenografts **A.** A schematic diagram showing the experimental procedure of P1 to P5 LnNE xenografts and primary cultured LnNE cells. LnNE xenografts were established as described in Material and Method. **B.** tumor take, **C.** tumor doubling time (average duration of tumor volumes that increase from 200 to 400 and from 400 to 800 mm^3^) and **D.** tumor growth curve were measured. **E.** Serum SYP concentration were measured 6 weeks after the tumor volumes reached 200mm^3^. **F.** Immunohistochemistry detected SYP, AR and PSA on LNCaP, and LnNE P1-P5 xenografts. Scale bars = 100μm.

To determine whether long-term androgen depletion can further promote NEPC progression of LnNE P0 xenografts, we inoculated LnNE P0 cells into castrated nude mice. Once the tumor volume reached 1000 mm^3^, xenografts were harvested. About 100mm^3^ tumor trunks were minced and re-inoculated into castrated mice to generate the next passage xenografts (Figure 2A). These experiments were repeated 5 cycles to obtain P1-P5 passages of LnNE tumors. The rest of the tissue samples from each passage were used for immunohistochemistry and primary culture to monitor cell morphology and neuroendocrine marker expression changes.

In the castrated mice where LNCaP xenografts do not form, the tumor take of LnNE P1 was 13 weeks but progressively reduced to 3.5 weeks at the P5 passage (Figure 2B). Tumor doubling times were also reduced from 3.3 weeks at P1 to 2.3 weeks at P5 (Figure 2C-2D). In addition, serum SYP concentrations increased in correlation with the LnNE tumor volumes (Figure 2E). Immunohistochemistry showed that SYP was negative in the LNCaP xenografts from none-castrated mice, but became positive in LnNE P1 with increased intensity in LnNE P4 and P5 tumors (Figure 2F). The AR remained positive in all tumors. However, AR negative cell population increased in LnNE P4 and P5 tumors. PSA expression was significantly reduced starting in LnNE P1 tumors, but was totally abolished in LnNE P3-P5 tumors. These results indicate that LnNE xenografts are progressively transformed into NEPC under long-term androgen depletion.

### Morphology and neuroendocrine phenotypes of LnNE cells

**Figure 3:**
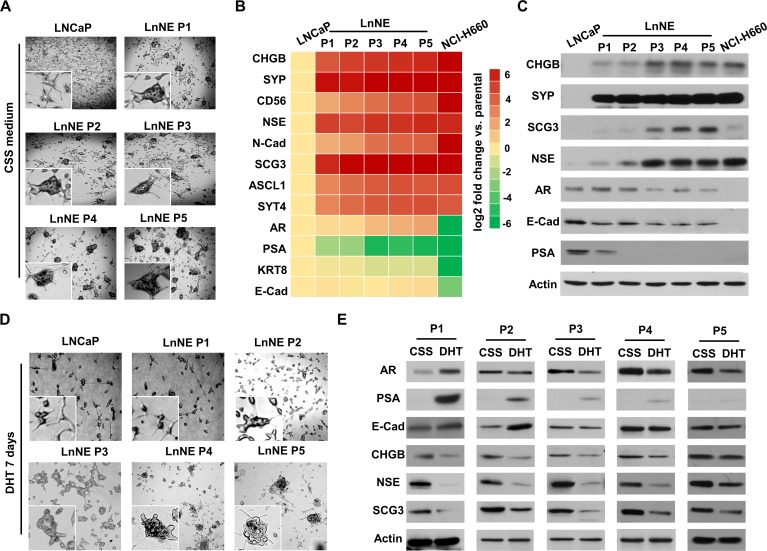
LnNE cell morphology and neuroendocrine phenotypes LNCaP and LnNE cell series P1 to P5 were cultured in the RPMI1640 medium containing 10% CSS and NCI-H660 cells were cultured in the HITES medium as described in the Material and Method for 2 weeks. **A.** Cell morphology changes were examined by the Zeiss fluorescent microscope. **B.**-**C.** Total RNA and protein lysates were collected and used to measure the expressions of neuroendocrine and AdPC biomarkers by real-time qPCR (B) and immunoblotting (C). LnNE cell series were cultured in the medium containing 10% CSS for 2 weeks followed by vehicle or 10nM DHT treatment for another week. **D.** Images of spheroid morphology were captured by the Zeiss fluorescent microscope. **E.** Whole cell protein lysates were collected and used to measure expressions of neuroendocrine and AdPC biomarkers by immunoblotting.

LNCaP cells cultured in the androgen depleted medium present with the epithelial spear morphology with compact cell bodies and extended fine branches while grow as adherent monolayers (Figure 3A). In contrast, LnNE P1-P5 cells formed 3-dimensional (3D) multicellular spheroids even under the 2-dimentional (2D) culturing conditions. These cells strongly expressed multiple neuroendocrine makers at both mRNA and proteins levels, similar to those of the well characterized NEPC cell line, NCI-H660 (Figure 3B-3C). It is worth to mention that there were dramatic inductions of CHGB, SCG3 and NSE expressions between P2 and P3 cells, implying that from the P3 passage on, LnNE cell/xenografts models have developed full and sustainable neuroendocrine phenotypes. Additionally, PSA expression disappeared at the P2 stage, while AR expression was reduced and kept in low levels in the LnNE P3-P5 passages (Figure 3C), indicating that AR transcriptional activity was abolished and the AR expression could be sustained at low levels during NEPC progression. These findings resemble many clinical NEPC tumors that show both AR and neuroendocrine maker positivity.

DHT treatment to P1 and P2 cells resulted in destruction of multicellular spheroid formation (Figure 3D). Cells became adherent to the surface of culturing dish and grew as monolayers similar to LNCaP cells. However, the morphology of LnNE P3, P4 and P5 cells remained in spheroids even in the presence of DHT. Our previous studies reported that the neuroendocrine phenotype of LnNE P0 cells could be reversed when treated with androgens [4]. In this study, we report that cells from early LnNE passages were still responsive to DHT, resulting in the upregulation of AR, PSA and E-Cad protein levels but downregulation of CHGB, SCG3 and NSE expressions (Figure 3E). Once reached to the P5 stage, however, LnNE cells became indifferent to DHT treatment with regard to morphology and biomarker expressions. In summary, we observed NEPC progression of the LnNE model from P0 to P5 under the prolonged castration condition, with the AR signaling diminishing at the P2 stage, neuroendocrine phenotypes fully developed at the P3 stage, and morphology and AdPC/neuroendocrine marker expressions being stabilized at the AR-indifferent P5 stage.

### Cell proliferation rate and transfection efficacy of LnNE cells

**Figure 4:**
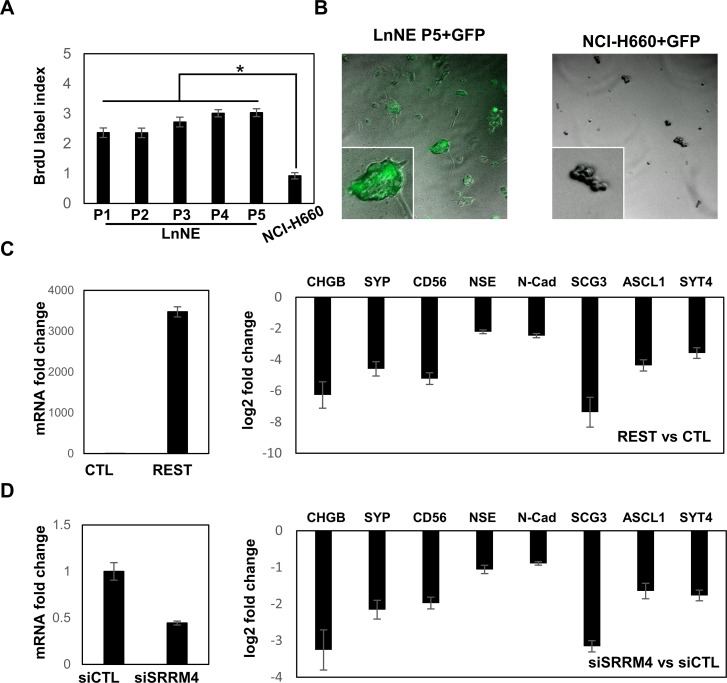
LnNE cell proliferation and transfection efficacy **A.** BrdU incorporation assays were performed on LnNE P1 to P5 cells and NCI-H660 cells. BrdU incorporation rates were calculated as described in the materials and methods. **B.** LnNE P5 and NCI-H660 cells were transfected with a GFP expression vector. Fluorescence images were captured by the Zeiss fluorescent microscope 24 hours after transfection. **C.**-**D.** LnNE P5 cells were transfected with (C) REST expression vector or (D) control or siRNA against SRRM4. Total RNA were collected and used to measure NEPC biomarkers expression by real-time PCR.

Using BrDU incorporation assays, LnNE cells cultured in the androgen depletion medium have a proliferation rate 2-3-fold higher than NCHI-H660 cells (Figure 4A). Importantly, the proliferation rate of LnNE cells increased with passage numbers, indicating that the LnNE cells have adapted androgen depletion conditions and gradually gained proliferative abilities in addition to stabilized NEPC biomarker expressions. These observations suggest the concurrence of transdifferentiation and accelerated proliferation during LnNE P0 to P5 progression. Moreover, when transfected with the GFP expression vector by lipofectamine 3000, the transfection efficacy was ∼50-70% in LnNE P5 cells but <2% in HCI-N660 cells (Figure 4B). We also transfected plasmid DNA encoding REST in LnNE P5 cells, resulting in ∼3500-fold induction of REST mRNA levels and ∼4-164-fold reduction of mRNA levels of neuroendocrine markers (Figure 4C). In addition, LnNE P5 cells can be transfected with the siRNA oligo, resulting in 56% depletion of SRRM4 mRNA and 1.8-9.5-fold reduction of mRNA levels of neuroendocrine markers (Figure 4D). These results indicate that LnNE cells have a relative high proliferation rate in vitro and can be transfected with plasmid DNA and siRNA to manipulate gene expression.

### *In vitro* LnNE spheroids for drug screening

**Figure 5:**
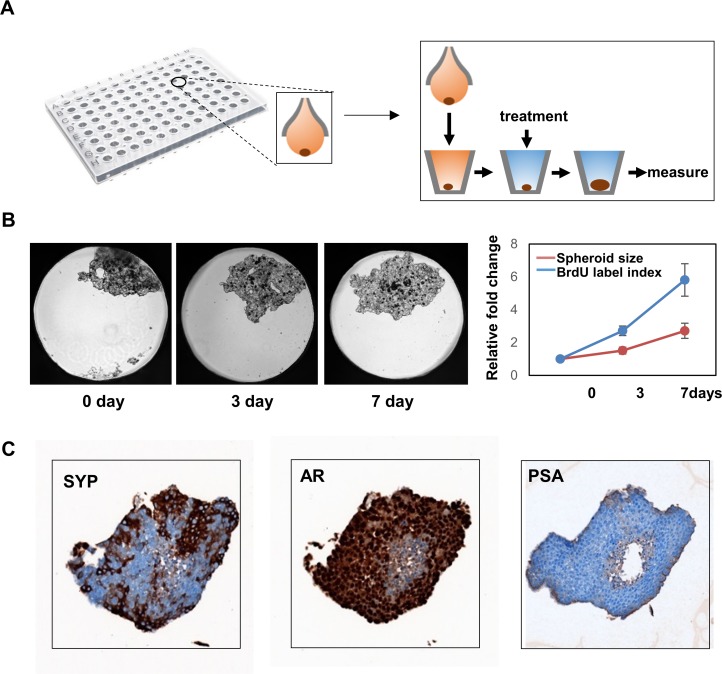
3-D culture of LnNE cells **A.** LnNE P5 cells were cultured to form 3D multicellular spheroids as described in Material and Method. **B.** Time-lapse images of spheroids were shown. Relative spheroid sizes and BrDU incorporation rates were measured at indicated time points. **C.** Spheroids were collected and paraffin embedded. Immunohistochemistry was performed using indicated antibodies.

NCI-H660 cells proliferate extremely slow in in vitro culture conditions, but grow rapidly as tumors in mice, implying that NEPC cells favor 3D culturing conditions. This is consistent to our observation that the LnNE cells formed spheroids even under the 2D culturing conditions (Figure 3). We applied the GravityPLUS Hanging Drop system (InSphero, Brunswick, USA) in a 96-well format to allow LnNE cell growth in 3D conditions (Figure 5A). LnNE P5 cells form single spheroid per well in ∼5 days, and the spheroids retained their morphology on flat-bottom 96-well plates for up to 7 days, during which the spheroid proliferation rate can be measured by the spheroid size as well as by BrDU incorporation assays (Figure 5B). Additionally, the spheroids can also be collected and paraffin embedded to perform immunohistochemistry (Figure 5C). We showed that LnNE P5 spheroids expressed high levels of SYP and AR but no PSA, consistent to the expression profiles of LnNE xenografts shown in Figure 2. These results indicate that multiple LnNE spheroids can be established in 96 well plates simultaneously to allow high throughput screening assays to identify potential small molecules that may inhibit NEPC cell proliferation or modulate NEPC cell morphology.

## DISCUSSION

We demonstrate that the LnNE can form multicellular spheroids under 2D or 3D culture conditions in vitro as well as xenografts in vivo. LnNE has global transcription and RNA splicing signatures similar to those of NEPC tumors. LnNE tumors are castrate-resistant and aggressively growing in nude mice. Although the AR protein expression remains, its ligand-dependent transcriptional activity on PSA secretion is deactivated and the neuroendocrine phenotype of LnNE cells cannot be reversed by androgen re-administration. We report that LnNE cells are relative highly proliferative, easy to be transfected and are suitable to perform high throughput screening assays.

The LnNE model is generated by introducing exogenous SRRM4 in adenocarcinoma cells. We have previously shown that SRRM4 can confer AdPC cells neuroendocrine phenotypes through compromising the function of genes such as REST and FOXA1 [4, 19]. Other SRRM4 target genes such as PTPRF and PTK2, have known functions in regulating cell proliferation or apoptosis [4], suggesting that these genes may enable cancer cell to gain growth and survival advantages under chemo- or hormonal therapies. It should also be noted that several SRRM4 target genes are histone acetyltransferases or de-methyltransferase (e.g. MEAF6 and BHC80) that may promote NEPC progression through epigenetic mechanisms [20]. Therefore, generation of the LnNE model suggest that NEPC can evolve directly from AdPC under the control of SRRM4. Through regulating RNA splicing and epigenetic mechanisms, SRRM4 induces two cellular processes including neuroendocrine differentiation and accelerated proliferation that collaboratively contribute to NEPC establishment. In addition, the phenotypical transitions of LnNE P0 to P5 support that NEPC progression is a gradual and chronical process. Neuroendocrine differentiation of cancer cells is initially transient and reversible, but becomes permanent possibly by NEPC-specific factors that promote cell proliferation. It is likely that multiple cycles of differentiation and proliferation are required for establishing NEPC.

SRRM4 strongly induces multiple neuroendocrine markers, among which SYP, CD56 and chromogranins are commonly used for NEPC diagnosis by pathologists. However, a challenge in early NEPC detection is the intra-tumoral heterogeneity as NEPC tumors do not show universal neuroendocrine marker profiles. Our primary results suggest that SRRM4 may be a more reliable diagnostic marker for NEPC than those previously reported because our immunohistochemistry on tissue microarray studies show that SRRM4 has a higher negative predictive value in ruling out NEPC tumors, and SRRM4 positivity has higher sensitivity than CD56, SYP and CHGA individually to NEPC.

The LnNE model also bears TP53 depletion, raising the question on whether loss-of-function of TP53 contributes to NEPC progression through either its canonical pathways that regulate cell cycling and apoptosis [21], or through promoting cell lineage plasticity mediated by Sox2 or Sox 11 [22–24]. Inactivation of TP53 was more frequent in NEPC (67%) compared to AdPC (31.4-53.3%) [3, 25, 26]. It is worth to be mention that the upregulation of basal and NE markers in the presence of anti-androgens only occurred when both TP53 and Rb1 were depleted. This is consistent to our previous results that showed no significant increase of neuroendocrine markers with TP53 inactivation alone [4]. Our GSEA analyses did not show further enhancement of NEPC gene signature when TP53 depletion was added to SRRM4 overexpression (Supplementary Figure 1B). In fact, our LnNE model has the RB1 gene intact and very low Sox2 and Sox11 mRNA levels. Since TP53 depletion enhances cell proliferation and colony formation of LNCaP cells overexpressing SRRM4 [4], we propose that loss-of-function of TP53 in the context of SRRM4 overexpression contributes to NEPC progression through enhancing cell proliferation.

It has been shown that AR protein expression can be detected by immunohistochemistry in NEPC, but the mRNA levels of AR target genes is significantly reduced [3]. Consistently, the LnNE model preserves AR protein levels but becomes PSA negative, supporting the notion that reduced AR function is concurrent with NEPC progression from adenocarcinoma with neuroendocrine differentiation to small cell neuroendocrine carcinoma [27]. Three other independent reports also showed that 27% (6/22), 38% (23/61) and 47% (7/15) of small cell neuroendocrine prostate tumors were AR protein positive, and in rarer instances, positive for AR target gene expressions including PSA and NKX3.1 [28–30]. These findings, together, suggest that remained AR expression and reduced/abrogated AR functions may be a common pathological characteristic in tumors with ongoing NEPC progression or in subsets of NEPC tumors. As we observed that our LnNE model had gradually reduced AR signaling as well as AR positive cell numbers during P0 to P5 xenograft passaging, it is therefore important to continue monitor AR expression and function as this model is grafted in castrated mice for higher passages.

In summary, we report an NEPC cell/xenograft model that has similar molecular and pathological features to those of NEPC tumors from patient samples. This model could be a useful research tool to study this disease.

## MATERIALS AND METHODS

### Cell lines and cell culture

The LNCaP and NCI-H660 cell lines were purchased from ATCC (Manassas, VA, USA). LnNE P0 cells are LNCaP cells with SRRM4 overexpression and TP53 knockdown that were described previously [4]. LNCaP cell was cultured in RPMI1640 medium with 10% FBS whereas LnNE P0 to P5 cells were maintained in phenol-free RPMI1640 medium with 10% charcoal-stripped serum (CSS) (Hyclone, Logan, UT, USA). NCI-H660 was cultured in HITES medium (RPMI1640 medium plus 0.005mg/ml Insulin, 0.01mg/ml Transferrin, 30nM Sodium selenite, 10nM Hydrocortisone, 10nM beta-estradiol and 2mM L-glutamine) with 10% FBS.

### RNA sequencing and bioinformatics analysis

RNA-seq data on both the VPC and Beltran 2016 cohorts were reported previously [3, 10]. Total RNA was extracted from LNCaP, LNCaP(SRRM4) and LnNE P0 cells using the mirVana Isolation Kit (Ambion Cat#:AM1560). After ribosomal RNA depletion, RNA sequencing was performed using Illumina HiSeq 2000 at the BGI Genomics (Shenzhen, China) according to standard protocols. RNA-Seq reads were mapped with Star (23104886) using Ensembl gene annotations GRCh38.87. Transcriptomes and splicing signatures were compared between samples using the Spearman correlation and hierarchical clustering with the pheatmap package in R. Gene Set Enrichment Analyses were performed by the GSEA software.

### Subcutaneous xenografts and primary cell culture

Male nude mice underwent castration surgery one week before 2×10^6^ LnNE P0 cells were inoculated subcutaneously. When xenografts appeared, tumor volumes were measured weekly. Once the tumor volume reaches 1000 mm^3^, xenografts were harvested. About 100mm3 tumor trunks were minced and re-inoculated into castrated mice to generate the next passage of the xenografts. These rest of the tissues were used for immunohistochemistry as well as primary cultures using the RPMI1640 medium plus 10% charcoal stripped serum to select adherent LnNE tumor cells. Mice serum was collected 6 weeks after the tumors reached to the size of 200mm^3^. Tumor doubling time was calculated by the average duration when tumor growth from 200-400mm3 and 400-800mm^3^. All animal experiments followed the protocol approved by the Institutional Animal Care and Use Committee at the University of British Columbia.

### Immunohistochemistry, real-time PCR and Western blotting assays

Immunohistochemistry, real-time PCR and Western blotting assays were described previously [4]. Information on antibody and primers was listed in Supplementary Materials. Real-time PCR and Western blotting experiments were repeated in three independent experiments and representative results were presented.

### Cell proliferation assay

Cell proliferation was measured by bromodeoxyuridine(BrdU) assay (Millipore, Cat#: 2750) according to the manufacturer's instructions. Cells were labeled by BrdU, which was further detected by anti-BrdU antibody. BrdU index was calculated as relative fold change in 450nm OD compared with the control group.

### Transient transfection

LnNE P5 and NCI-H660 cells were transfected with GFP expression vectors using the Lipofactamine 3000 transfection reagents according to the provided protocols. Transfection efficacy was detected by GFP protein expression level 24 hours after transfection. Cell imaging was captured by Zeiss fluorescent microscope (Carl Zeiss, Thornwood, NY).

### 3D spheroid culture

The 3D spheroids of LnNE cells were generated using the GravityPLUS™ Hanging Drop System according to the manufacturer's protocol. The growth medium was replenished every other day. After spheroid formation, the micro-tissue spheroids were transferred into the GravityTRAP™ culture plates for further experiments. Images of spheroids were captured by Zeiss fluorescent microscope (Carl Zeiss, Thornwood, NY) and the sizes of the spheroids were analyzed by Imagine-J software.

## SUPPLEMENTARY MATERIALS FIGURE AND TABLES


