# Supplementary material for: Establishment of a neuroendocrine prostate cancer model driven by the RNA splicing factor SRRM4
Source: Oncotarget. 2017 Aug 3;8(40):66878–88. doi: 10.18632/oncotarget.19916 (PMC5620142; doi:10.18632/oncotarget.19916)
Supplement: Supplementary file 1 [file oncotarget-08-66878-s001.pdf]

Establishment of a neuroendocrine prostate cancer model driven by the RNA splicing factor SRRM4

SUPPLEMENTARY MATERIALS

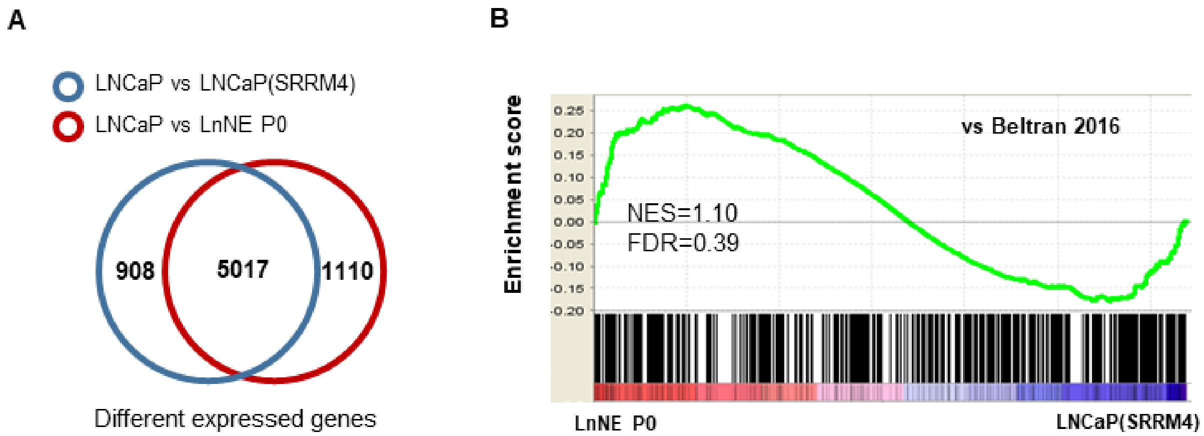

Supplementary Figure 1: (A) A Venn-diagram representation of RNA-seq results comparing the differentially expressed genes (adjusted p-value < 0.01) between LNCaP vs LNCaP(SRRM4) and LNCaP vs LnNE P0 cells**.** (B) GSEA results compareing the LNCaP(SRRM4) and LnNE P0 expression profiles with respect to the NEPC-specific transcriptome from Beltran 2016 data set.

Supplementary Table 1: Reagents information

Name	Cat No.	Company
DHT	1940	Cedarlane

Supplementary Table 2: Real time qPCR primers information

GENE	Sequence
REST F:	TGCGTACTCATTCAGGTGAGA
REST R:	TCTTGCATGGCGGGTTACTT
SYP F:	GGCCCTTTGTTATTCTCTCGGTA
SYP R:	GGCCATTTCTGAGGCTAAACT
CHGB F:	CGAGGGGAAGATAGCAGTGAA
CHGB R:	CAGCATGTGTTTCCGATCTGG
SYT4 F:	ATGGGATACCCTACACCCAAAT
SYT4 R:	TCCCGAGAGAGGAATTAGAACTT
ASCL1 F:	CCCAAGCAAGTCAAGCGACA
ASCL1 R:	AAGCCGCTGAAGTTGAGCC
SCG3 F:	GTCTTCATCAACTAGACGGGACT
SCG3 R:	ACAATCTTGTCAAACACGGCTC
NSE F:	CCGGGAACTCAGACCTCATC
NSE R:	CTCTGCACCTAGTCGCATGG
18s rRNA F:	TTGACGGAAGGGCACCACCAG
18s rRNA R:	GCACCACCACCCACGGAATCG
N-Cad F:	TGCGGTACAGTGTAACTGGG
N-Cad R:	GAAACCGGGCTATCTGCTCG
CD56 F:	GGCATTTACAAGTGTGTGGTTAC
CD56 R:	TTGGCGCATTCTTGAACATGA
KRT8 F:	TCCTCAGGCAGCTATATGAAGAG
KRT8 R:	GGTTGGCAATATCCTCGTACTGT
PSA F:	AGTGCGAGAAGCATTCCCAAC
PSA R:	CCAGCAAGATCACGCTTTTGTT

Supplementary Table 3: Antibody information

Name	Description	Cat No.	Company
Actin		A2066	Sigma
Flag-tag	M5	F4042	Sigma
CHGB	C-19	sc-1489	Santa Cruze
SYP	D-4	sc-17750	Santa Cruze
NSE		MAB324	Millipore
SCG3	C-19	sc-1492	Santa Cruze
AR	N-20	sc-816	Santa Cruze
E-cadherin	H-108	sc-7870	Santa Cruze
PSA	C-19	sc-7638	Santa Cruze

Supplementary Table 4: siRNA information

Name	Cat No.	Company
siSRRM4	L-019322-02-0005	Dharmacon
